# A comparative study of smooth muscle cell characteristics and myofibroblasts in processus vaginalis of pediatric inguinal hernia, hydrocele and undescended testis

**DOI:** 10.1186/s12894-024-01449-0

**Published:** 2024-05-30

**Authors:** Nellai Krishnan, Aanchal Kakkar, Tapas Chandra Nag, Sandeep Agarwala, Prabudh Goel, Anjan Kumar Dhua

**Affiliations:** 1 Department of Paediatric Surgery, All India Institute of Medical Sciences, New Delhi, India; 2 Department of Pathology, All India Institute of Medical Sciences, New Delhi, India; 3 Department of Anatomy, All India Institute of Medical Sciences, New Delhi, India

**Keywords:** Smooth muscle, Myofibroblasts, Processus vaginalis, Electron microscopy, Immunohistochemistry, Inguinal hernia, Hydrocele, Undescended testis

## Abstract

**Background:**

Congenital inguinal hernia, hydrocele and undescended testis (UDT) are associated with patent processus vaginalis. The smooth muscles present in the processus vaginalis aid in the descent of the testis and undergo programmed cell death after testicular descent leading to obliteration. The persisting amount of smooth muscle in the processus vaginalis influences the clinical outcome as inguinal hernia, hydrocele or UDT. Therefore, a study was conducted to evaluate the processus vaginalis in these three conditions to observe the presence and phenotype of smooth muscle cells and the presence of myofibroblasts.

**Materials and methods:**

The processus vaginalis sacs in patients with inguinal hernia, hydrocele and UDT were examined using light microscopy for the presence and distribution of smooth muscle cells and immunohistochemical staining for vimentin, desmin, and α-smooth muscle actin (SMA) to identify the smooth muscle phenotype. Transmission electron microscopy was also performed in all the sacs to observe the presence of myofibroblasts.

**Results:**

Seventy-eight specimens of processus vaginalis (from seventy-four patients), distributed as 47%, 27%, and 26% as inguinal hernia, hydrocele and UDT respectively, were included in the study. The sacs from inguinal hernia and hydrocele had significantly more presence of smooth muscles distributed as multiple smooth muscle bundles (*p* < 0.001). Desmin and SMA staining of smooth muscle cells was observed in significantly more sacs from hydrocele, followed by inguinal hernia and UDT (*p* < 0.001). The sacs from UDT had a significant presence of striated muscles (*p* = 0.028). The sacs from inguinal hernia had a significant presence of myofibroblasts, followed by hydrocele and UDT (*p* < 0.001) and this significantly correlated with the light microscopy and immunohistochemical features. The processus vaginalis sacs from four female patients did not differ statistically from the male inguinal hernia sacs in any of the above parameters.

**Conclusion:**

The processus vaginalis sacs in pediatric inguinal hernia, hydrocele and undescended testis differ in the presence, distribution and phenotype of smooth muscles and the presence of myofibroblasts. The clinical presentations in these entities reflect these differences.

## Introduction

Inguinal hernia, undescended testis (UDT) and hydrocele are common conditions that need pediatric surgical consultation during childhood. The overall incidence of inguinal hernias is 5% for males [1]. Inguinal hernia is 5 to 10 times more common in males than females. Bilateral inguinal hernias are seen in 10% of patients. Undescended testis occurs in approximately 3% of term male infants but in preterm, the incidence rises to 33–45%. Most testes descend within the first 6–12 months and the incidence of undescended testis decreases to 1% at one year. Congenital hydrocele typically resolves by two years of age, so surgery is not recommended in the first 2 years of life unless the hydrocele is communicating. Hydroceles that persist beyond 2 years of age require operation.

Congenital inguinal hernia and hydrocele are associated with patent processus vaginalis. Although the processus vaginalis is also patent in children with undescended testis, clinical inguinal hernia is rare and is encountered in approximately 10-15% of cases [2]. The processus vaginalis is an evagination of the peritoneum through the deep inguinal ring. It can first be identified during the third month of gestation [3]. The intra-abdominal testis passes through the processus vaginalis during the inguinoscrotal phase of testicular descent in the seventh to ninth months of gestation. Following this, the portion of processus vaginalis lying above the testicle obliterates, thereby closing the internal inguinal ring. In females, the canal of Nuck corresponds to the processus vaginalis and it communicates with the labia majora. The canal of Nuck closes approximately at the seventh month of gestation.

The genitofemoral nerve (GFN) and calcitonin gene–related protein (CGRP) has been implicated in both testicular descent and obliteration of the processus vaginalis [4, 5]. Reduced CGRP release from the GFN prenatally may result in undescended testis, whereas reduced CGRP postnatally may lead to hernias and hydroceles. The physical force for the descent of the testis is provided by the intra-abdominal pressure and the propulsive force generated by the muscles derived from the gubernaculum. Since the obliterated processus vaginalis is devoid of smooth muscle, the obliteration of the processus vaginalis is associated with disappearance of smooth muscle by programmed cell death [2, 6, 7]. The smooth muscle cells undergo programmed cell death after the descent of the testis resulting in the closure of the processus vaginalis. Dedifferentiation of the smooth muscle cells into myofibroblasts is an important step in this transformation. Myofibroblasts may represent their attempts to disappear through apoptosis. The accurate detection of myofibroblasts requires electron microscopy as their cytoskeletal phenotypes vary and immunohistochemistry may not be adequate to detect them [8]. Few studies have shown that the persisting amount of smooth muscle in the processus vaginalis influences the clinical outcome as inguinal hernia, hydrocele or undescended testis [2, 6, 9, 10].

The smooth muscle cells at various stages of maturity stain differently with the immunohistochemical markers such as desmin, α-smooth muscle actin (SMA) and vimentin. Desmin is an intermediate filament present in smooth muscle, vimentin is seen in mesenchymal tissue and SMA is present in myofibroblasts. Mature smooth muscle cells, which are highly contractile, fully differentiated phenotypes, are characterized by a well-developed system of contractile myofilaments, instead of synthetic organelles. These cells express desmin and SMA. Immature smooth muscle cells, which are of synthetic phenotype, are characterized by well-developed synthetic organelles, especially Golgi apparatus, and reduced contractile myofilaments. These cells express high amounts of vimentin but low amounts of desmin and SMA. Few studies have evaluated the diversity and differentiation of smooth muscle phenotypes in processus vaginalis through the expression of desmin, vimentin and SMA immunohistochemically [11, 12]. However, these studies have not examined the smooth muscle phenotypes in inguinal hernia, hydrocele and undescended testis concurrently.

Since the defective closure of processus vaginalis results leads to varied presentations in inguinal hernia, hydrocele and undescended testis; the histopathology, immunohistochemical features and electron microscopic examination would differ between these entities. Therefore, a study was planned to evaluate the processus vaginalis from inguinal hernia, hydrocele and undescended testis to elucidate the reason for the varied clinical presentation of defective closure of processus vaginalis in these three conditions to provide insight into the role of differentiation of smooth muscle cells.

## Materials and methods

The institute’s ethical clearance vide Ref No.: IECPG-237/22.04.2019 was taken and then patients were recruited. This was a single-centre cross-sectional study. The inclusion criteria were pediatric patients with inguinal hernia, hydrocele or undescended testis and pediatric patients undergoing laparotomy for various other pathologies were included as controls. The patients with associated conditions such as ventriculoperitoneal shunt, bladder exstrophy, and spinal defect were excluded. The patients with more than one pathology (inguinal hernia, hydrocele or undescended testis) on the same side or those undergoing laparoscopic surgery for inguinal hernia, or undescended testis were also excluded.

Sample size calculation was based on convenient sampling due to the logistics and cost involved. We planned to include at least 20 patients, each with an inguinal hernia, hydrocele and undescended testis. Peritoneum samples from five children who underwent laparotomy for various other indications were taken as negative controls. Histopathological examination, immunohistochemical staining and electron microscopy were done in all the specimens. Parental informed consent was taken for all the children enrolled in the study.

### Overview of study protocol

Herniotomy was performed after high ligation of the sac at the deep ring. In the case of bilateral pathologies, the sacs of each side were analysed independently. Peritoneal samples were taken from pediatric patients undergoing laparotomy or laparoscopy for various other indications to serve as controls. The excised processus vaginalis sac and peritoneal specimen were divided into two parts for further analysis, one each for histopathology and electron microscopy examination. The pathologist and anatomist evaluating the specimens were blinded regarding the diagnosis.

The first part was fixed in 10% neutral buffered formalin and sections from them were embedded in paraffin as done routinely. 4 μm thick paraffin sections were stained with haematoxylin and eosin. These were examined under light microscopy for the presence and distribution of smooth muscle cells and for the presence of myofibroblasts, if any. If present, the smooth muscle bundles were graded semi-quantitatively as few (dispersed) or multiple smooth muscle bundles.

Immunohistochemical markers: Unstained paraffin sections were used for immunohistochemical staining. Immunostaining for vimentin, desmin and α-smooth muscle actin (SMA) were performed. Samples were deparaffinized in xylene and rehydrated in graded ethanol solutions. Then, the sections were subjected to pre-treatment to enhance antigen retrieval. A standard streptavidin–biotin method was used. Finally, counterstaining with haematoxylin was performed. Samples were considered positive for desmin, vimentin, or SMA when specific intracytoplasmic and/or membranous staining was observed. The immunoreactivity of smooth muscle cells to all of the above-mentioned immunostains was evaluated. Positive and negative controls were used to assess the specificity of the staining.

Electron Microscopy: The second part of the tissue sample was fixed in Karnovsky fixative (2% glutaraldehyde and 4% paraformaldehyde) for 24 h, following which the sample was washed with phosphate buffer. Then post-fixation was done with osmium tetroxide in a phosphate buffer. After dehydrating in ethanol gradient at room temperature, tissue samples were embedded in epoxy resin. Ultra-thin Sect. (70 nm thick) were cut with ultramicrotome and copper grids were prepared. Then the sections were stained with uranyl acetate and lead citrate. The grids were placed in a specimen holder and observed in a Tecnai transmission electron microscope for the presence of myofibroblasts. Myofibroblasts are large spindle-shaped or stellate cells with several long cytoplasmic extensions, are enveloped in an external lamina and have distinct sub-plasmalemmal attachment plaques and are characterised by finely granular chromatin, prominent nucleoli, poorly developed Golgi apparatus and rough endoscopic reticulum. They represent an intermediate stage in differentiating smooth muscle cells into fibroblasts.

### Statistical analysis

Data entry was done using Microsoft® Excel® 2019 (Version 2306 Build16.0.16529.20164) 64-bit and statistical analysis was done using IBM Statistical Package for Social Sciences Statistics (Version 29.0.1). The groups were compared by One-way analysis of variance (ANOVA), χ2 (chi-square) test or Fisher’s exact test with a threshold of significance of *P* < 0.05.

## Results

### Patient characteristics

Seventy- four patients were included in the study. Out of these, four patients had bilateral pathology (two each had bilateral inguinal hernia and UDT). Therefore, seventy-eight patient specimens of processus vaginalis were included in the study. Out of these, thirty-seven (47%) were inguinal hernia, twenty-one (27%) were hydrocele and twenty (26%) specimens were UDT. This included four female patients with inguinal hernia. The mean age (months) of the patients in the three groups were 45 (± 37.7), 45.3 (± 29.8) and 44.1 (± 29.2) respectively and there was no statistical difference (*p* = 0.993, ANOVA) amongst the groups. Peritoneum samples (controls) were taken from five patients who underwent laparotomy for unrelated pathology. The control specimens showed the presence of fibro-collagenous tissue only.

### Histology and immunohistochemistry

Smooth muscles were observed in 28 (76%) specimens of inguinal hernia, 18 (86%) of hydrocele and 4 (20%) of UDT; p = < 0.001 (Table 1).

**Table 1:** Presence of smooth muscle in the processus vaginalis sac. UDT = undescended testis

		Smooth muscles (%)		
		No	Yes	p value
Diagnosis	Hernia	9 (24)	28 (76)	< 0.001 (chi-squared test)
	Hydrocele	3 (14)	18 (86)	
	UDT Total	16 (80) 28 (36)	4 (20) 50 (64)	

**Fig. 1:**
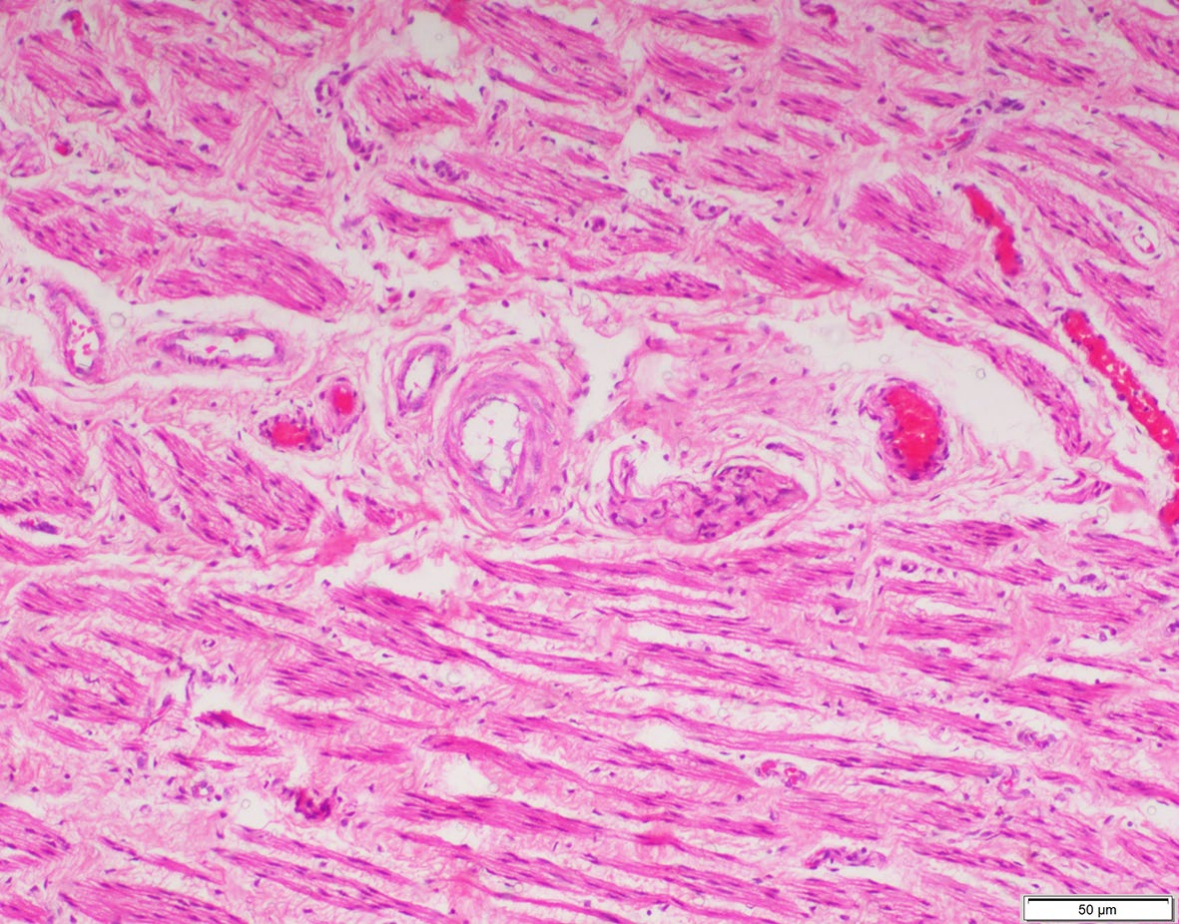
Multiple smooth muscle bundles observed in sacs of inguinal hernia. (H&E X100)

**Fig. 2:**
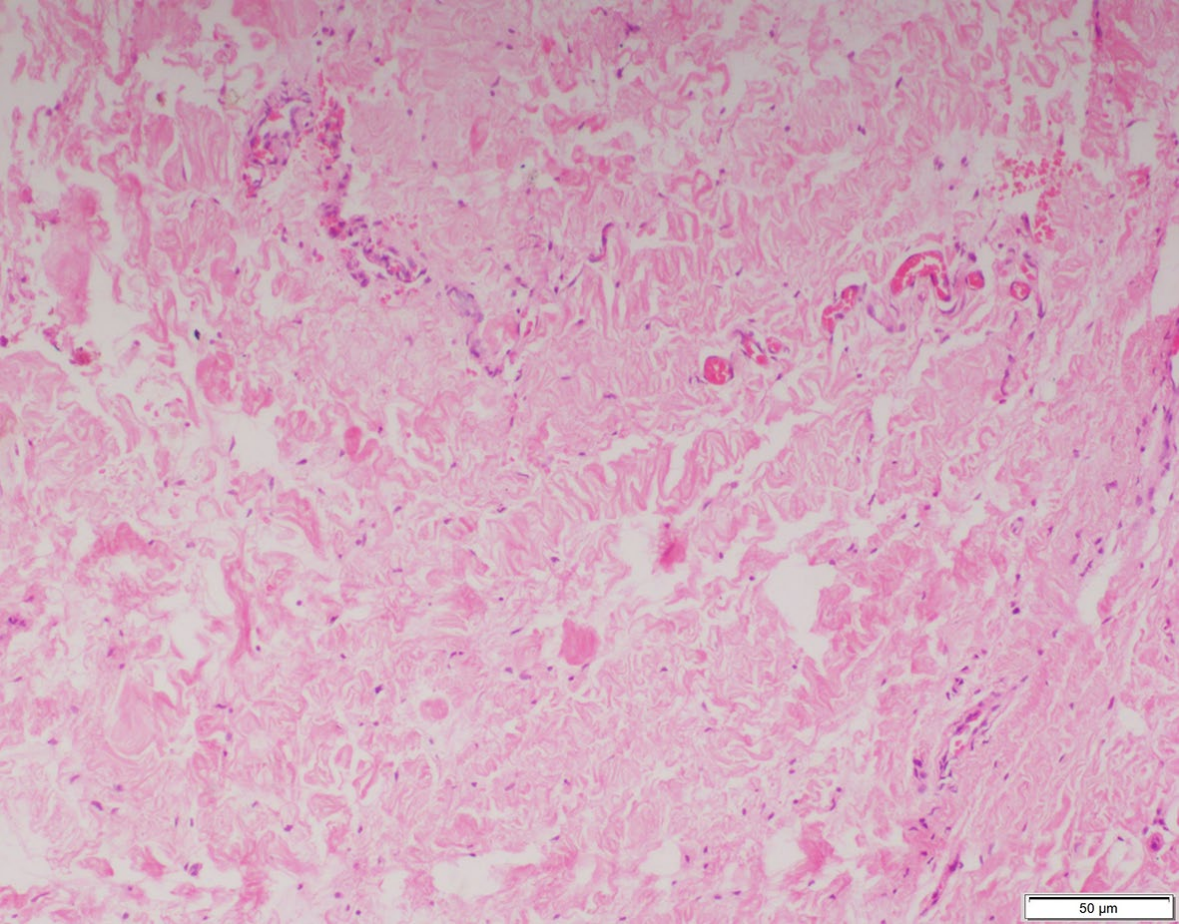
Few smooth muscle bundles observed in sacs of hydrocele. (H&E X100)

**Fig. 3:**
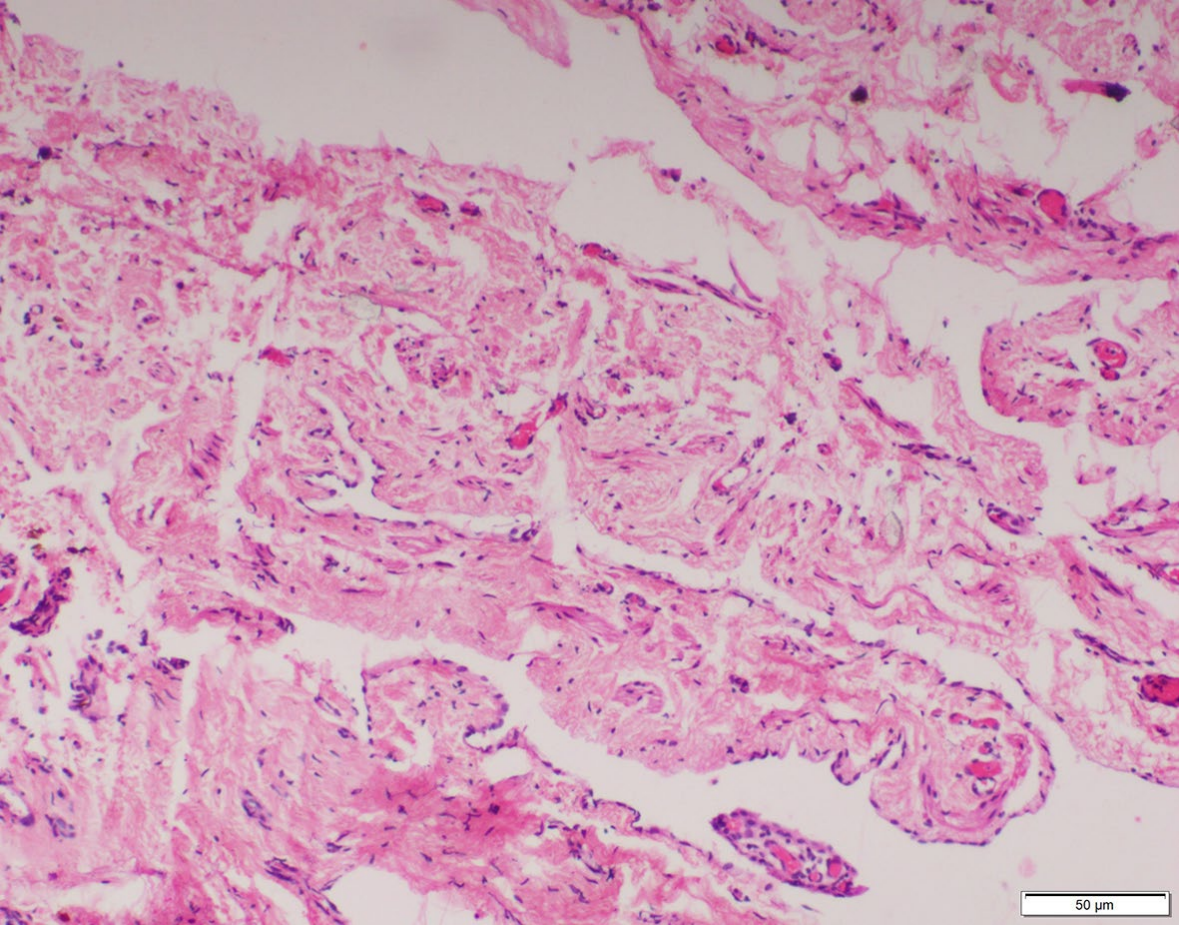
No smooth muscles observed in sacs of undescended testis. (H&E X100)

The smooth muscles were distributed as few and scattered in 15 (40%), 11 (52%) and 4 (20%) specimens of inguinal hernia, hydrocele and UDT respectively. Multiple smooth muscle bundles were observed in 13 (35%) and 7 (33%) specimens of inguinal hernia and hydrocele respectively (Figs. 1 and 2). None of the specimens of UDT showed multiple smooth muscle bundles (Fig. 3). These differences were statistically significant (*p* < 0.001) (Table 2).

**Table 2:** Distribution of smooth muscle bundles in the processus vaginalis sacs. UDT = undescended testis

			Smooth muscle bundles (%)			
			No	Few	Multiple	p value
Diagnosis	Hernia		9 (24)	15 (40)	13 (35)	< 0.001 (chi-squared test)
	Hydrocele		3 (14)	11 (52)	7 (33)	
	UDT		16 (80)	4 (20)	0	
Total			28 (36)	30 (38)	20 (25)	

**Fig. 4:**
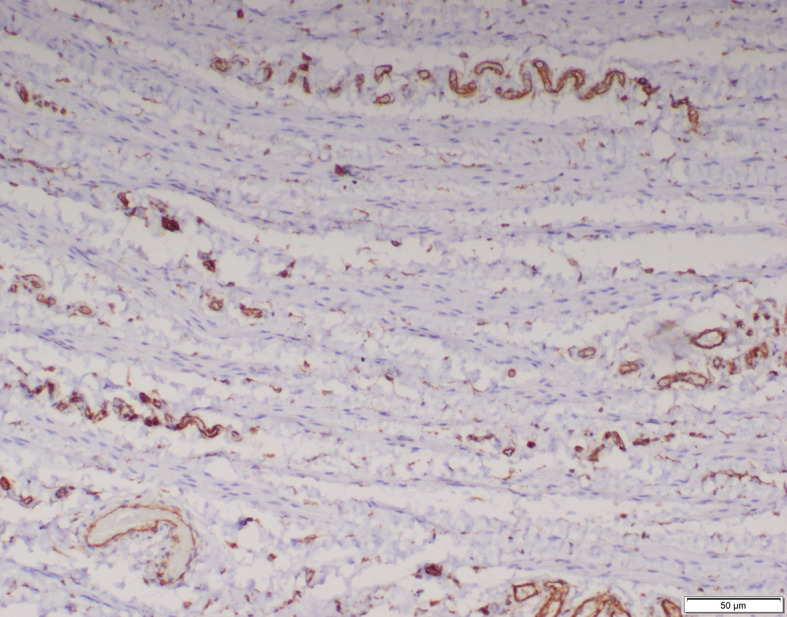
Smooth muscle cells on processus vaginalis negative for vimentin (x100)

**Fig. 5:**
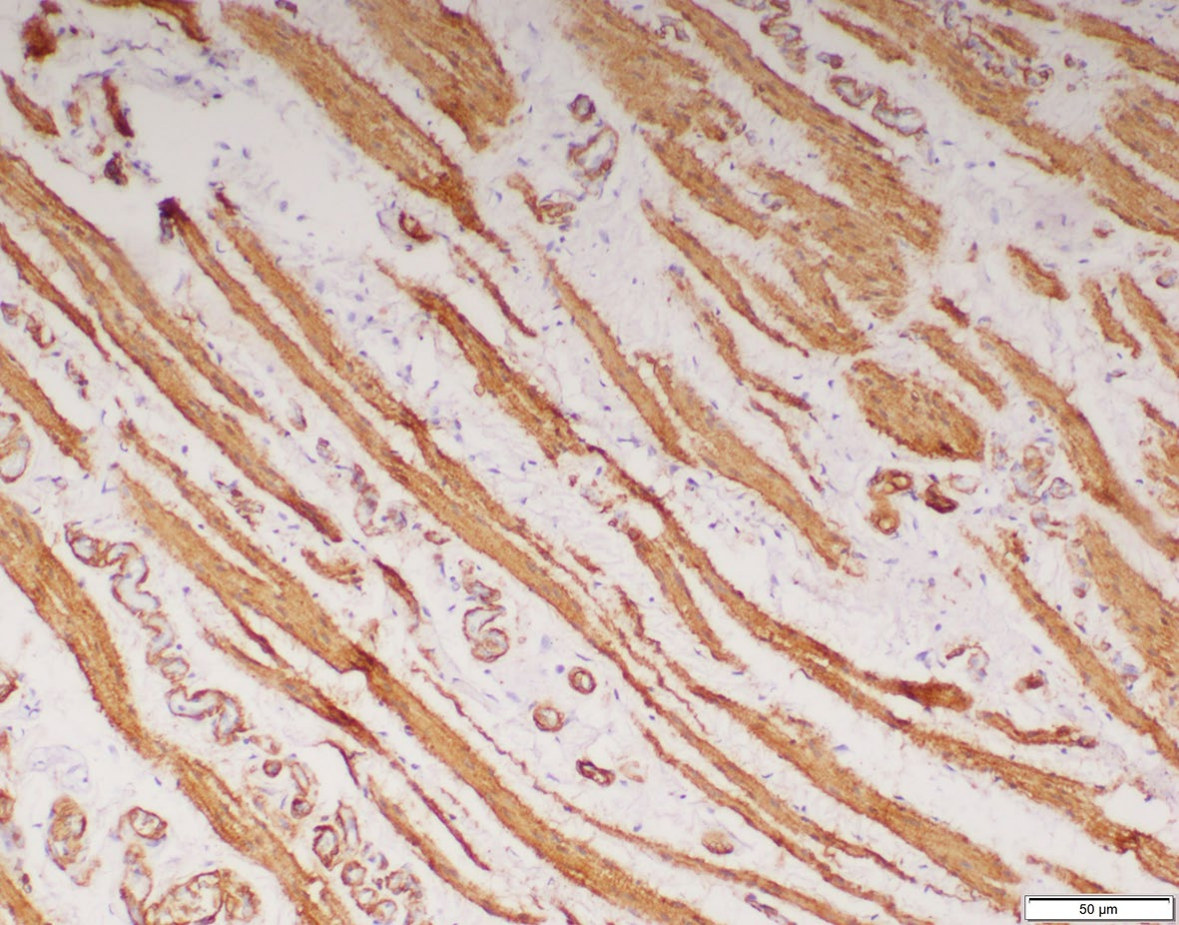
Smooth muscle cells on processus vaginalis positive for smooth muscle actin (x100)

**Fig. 6:**
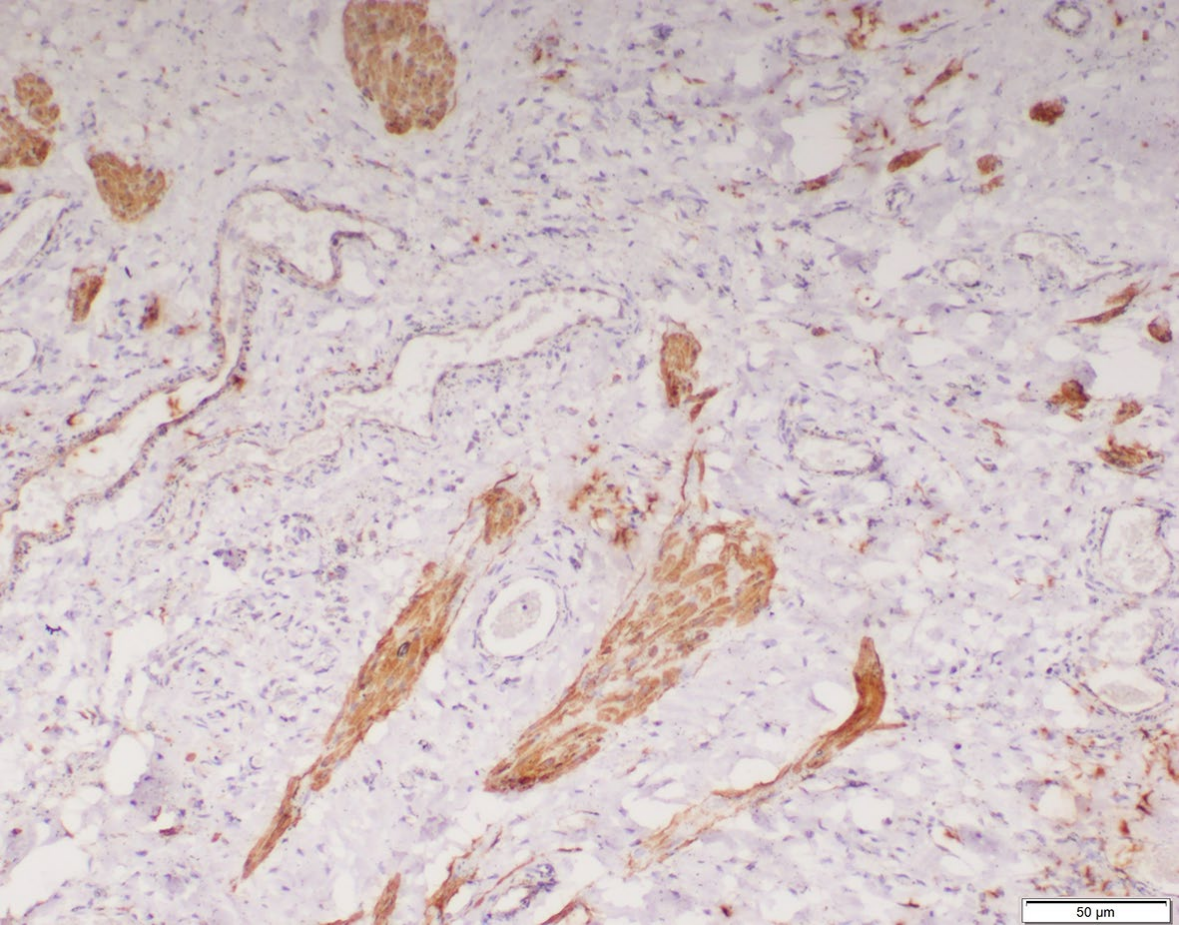
Smooth muscle cells on processus vaginalis positive for desmin (x100)

Immunohistochemical staining was conducted on the processus vaginalis sacs to evaluate the maturation of smooth muscle. None of the specimens of inguinal hernia, hydrocele and UDT showed any vimentin positive smooth muscle (Fig. 4). Two processus vaginalis sacs of inguinal hernia showed few scattered smooth muscles which were negative for both desmin and smooth muscle actin (SMA). One specimen of inguinal hernia was positive for desmin but negative for SMA and another specimen of inguinal hernia was positive for SMA but negative for desmin. Both of these specimens had few and scattered smooth muscles. In the rest of the specimens, smooth muscles when present showed positivity to both desmin and SMA (Figs. 5 and 6). Overall, positive staining for desmin and SMA was observed in 25 (68%), 18 (86%) and 4 (20%) specimens of inguinal hernia, hydrocele and UDT respectively; *p*<0.001 (Table 3).

**Table 3:** Smooth muscle actin expression in the processus vaginalis sacs. UDT = undescended testis

			Desmin and smooth muscle actin (%)		
			No	Yes	p value
Diagnosis	Hernia		12 (32)	25 (68)	< 0.001 (chi-squared test)
	Hydrocele		3 (14)	18 (86)	
	UDT		16 (80)	4 (20)	
Total			31 (40)	47 (60)	

Striated muscles were observed in the processus vaginalis sacs of 10 (27%), 1 (5%) and 8 (40%) specimens of inguinal hernia, hydrocele and UDT respectively and this difference was statistically significant (*p* = 0.028).

Myofibroblasts were noted in light microscopy in four specimens (two each of inguinal hernia and UDT). The immunohistochemical expression of the myofibroblasts in these four specimens were variable. Desmin positive and vimentin negative myofibroblasts were seen in the two UDT processus vaginalis specimens. However, one specimen of inguinal hernia showed desmin positive and vimentin positive myofibroblasts and another specimen of inguinal hernia showed desmin positive and smooth muscle actin positive myofibroblast. The accurate detection of myofibroblasts requires electron microscopy as their cytoskeletal phenotypes vary and immunohistochemistry may not be adequate to detect them. Therefore, electron microscopy was conducted in the processus vaginalis specimens and myofibroblasts were noted in a significantly greater number of specimens using electron microscopy (detailed subsequently).

### Transmission electron microscopy

**Fig. 7:**
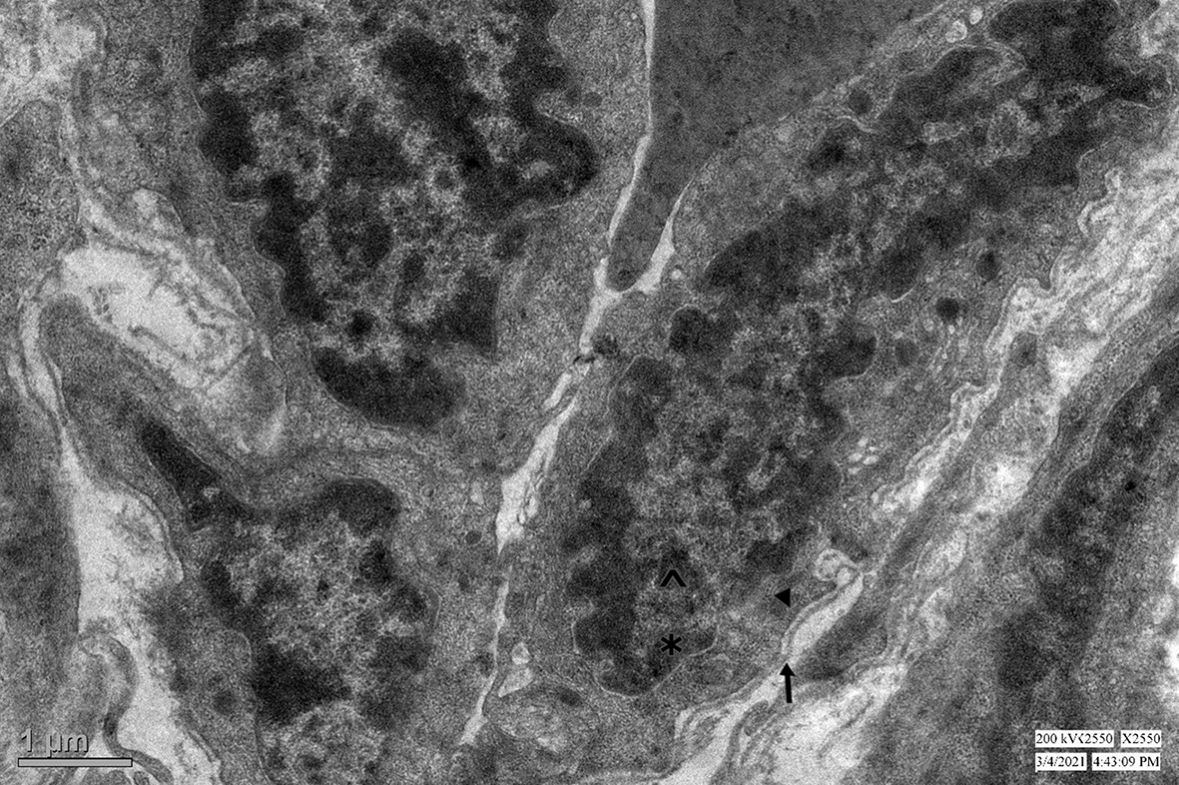
Transmission electron microscope showing myofibroblasts. These cells are enveloped in an external lamina (arrow) and have distinct sub-plasmalemmal attachment plaques (arrowhead), finely granular chromatin (asterisk) and prominent nucleoli (caret). (x2550)

Myofibroblasts are large cells, spindle shaped or stellate with several long cytoplasmic extensions, enveloped in an external lamina and have distinct sub-plasmalemmal attachment plaques, finely granular chromatin, prominent nucleoli and have poorly developed Golgi area and rough endoscopic reticulum (Fig. 7). The ultrastructural features of myofibroblasts were present in 35 (95%), 14 (67%) and 3 (15%) of processus vaginalis specimens of inguinal hernia, hydrocele and UDT respectively; *p* < 0.001 (Table 4).

**Table 4:** Presence of myofibroblasts in the processus vaginalis specimens identified using transmission electron microscopy. UDT = Undescended testis

			Myofibroblast (%)		
			No	Yes	p value
Diagnosis	Hernia		2 (5)	35 (95)	< 0.001 (chi-squared test)
	Hydrocele		7 (33)	14 (67)	
	UDT		17 (85)	3 (15)	
Total			26 (33)	52 (67)	

The presence of myofibroblasts on electron microscopy significantly correlated with the presence of smooth muscle in light microscopy and expression of desmin and smooth muscle actin on immunohistochemistry (Table 5)

**Table 5:** Correlation between the presence of myofibroblasts in electron microscopy and presence of smooth muscle in light microscopy and expression of desmin and smooth muscle actin on immunohistochemistry

Correlations				
		Smooth muscles	Desmin	Smooth muscle actin
Myofibroblast	Pearson Correlation	0.378	0.315	0.315
	Sig. (2-tailed)	< 0.001	0.005	0.005
	N	78	78	78

### Influence of gender on the processus vaginalis characteristics

The processus vaginalis sacs from four female patients with inguinal hernia did not differ statistically from the processus vaginalis sacs from thirty-three male patients with inguinal hernia with respect to presence of smooth muscles (*p* = 0.69), distribution of smooth muscle bundles (*p* = 0.146), expression of desmin (*p* = 0.609), smooth muscle actin (*p* = 0.609), presence of striated muscles (*p* = 0.052) and presence of myofibroblasts (*p* = 0.207).

## Discussion

The processus vaginalis is a diverticulum of the peritoneum, inside the gubernaculum. The gubernacular mesenchyme gives rise to smooth muscles, which aids in the descent of the testis into scrotum. The processus vaginalis obliterates after the descent of the testis and the obliterated processus vaginalis is devoid of smooth muscle cells. Inguinal hernia and hydrocele in children are closely associated with the presence of the smooth muscle bundles in processus vaginalis. The patent processus vaginalis in hydrocele seems to allow only the passage of fluid in contrast to inguinal hernia. The patent processus vaginalis in undescended testis usually is not associated with inguinal hernia or hydrocele. This suggests that the patent processus vaginalis differs in these three entities. Therefore, we evaluated the processus vaginalis sacs in these three pathologies using histopathology, immunohistochemistry and electron microscopy to understand the phenotypical differences in these conditions.

In our study, smooth muscles were present in 76% of inguinal hernia and 86% of hydrocele specimens, but only 20% of specimens of UDT had the presence of smooth muscles and this difference was statistically significant. Moreover, the smooth muscle cells were organised as multiple bundles in inguinal hernia and hydrocele specimens compared to UDT specimens, where the smooth muscles were sparse and scattered, if present. Our study partially concurs with the studies by Tanyel, et al. wherein the authors reported smooth muscle was present in inguinal hernia sacs but only sparse smooth muscles were seen in hydrocele and UDT sacs [6, 9]. Whereas, a study by Piçarro, et al. observed smooth muscles in 68% of the sacs in UDT cases [10]. In all these studies, the smooth muscle cells were present in fewer patients of UDT as compared to inguinal hernia. The apparent differences in the presence of smooth muscles might be due to the age of the patients enrolled in the study. The mean (SD) of the patients enrolled in our study were 45 (± 37.7), 45.3 (± 29.8) and 44.1 (± 29.2) in inguinal hernia, hydrocele and UDT, respectively. The mean ages of the patients in the study by Tanyel, et al. [9] and Piçarro, et al. [10] were both higher. This could suggest that although processus vaginalis persists in these three conditions, the processus undergoes changes as the child grows. The smooth muscle content increases when there is failure of obliteration of processus vaginalis.

Immunohistochemical staining was done to evaluate the smooth muscle phenotype in the processus vaginalis specimens. Our study showed positive staining for desmin and SMA in 68%, 86% and 20% of the processus vaginalis sac of inguinal hernia, hydrocele and UDT, respectively and this difference was statistically significant. There was no vimentin-positive smooth muscle in any specimen. This contrasts with the report by Mouravas, et al., wherein the authors reported that vimentin was expressed by all sacs of hydrocele and half of the inguinal hernia sacs [11]. Another study by the same centre reported that vimentin was expressed in more than 90% of the UDT specimens [12]. Since vimentin is not expressed by mature smooth muscle cells, therefore the smooth muscles in processus vaginalis identified in our study were of mature phenotype. In our study, although the quantity of smooth muscle cells decreased in sacs of UDT, there was no change in the smooth muscle phenotype. Therefore, the processus vaginalis in UDT specimens are deficient in smooth muscles. Since smooth muscles in the processus vaginalis contribute to the descent of the testis, therefore the deficient smooth muscle in these specimens might contribute to the disease pathology. As the obliteration of processus vaginalis starts after the descent of the testis, the de-differentiation and apoptosis of smooth muscle cells have not started in these sacs, as evident by the mature phenotypic staining.

Myofibroblasts are intermediary cells in the process of dedifferentiation of smooth muscle cells to fibroblasts. The presence of the myofibroblasts were noted in 95%, 67% and 15% of the processus vaginalis specimens of inguinal hernia, hydrocele specimens and UDT. The presence of myofibroblasts in the majority of the specimens of hernia and hydrocele supports the theory of de-differentiation of smooth muscle cells to fibroblasts. This concurs with the results by Tanyel et al., wherein myofibroblasts were observed in all sacs of the inguinal hernia [13]. However, there is no previous literature available regarding the presence of myofibroblasts in specimens of hydrocele and UDT. The scarce presence of myofibroblasts in UDT specimens suggests that de-differentiation has not yet started in the sacs of UDT, thereby indicating the processus vaginalis undergoes de-differentiation once the descent of testis is completed. The presence of myofibroblasts on electron microscopy significantly correlated with the presence of smooth muscle in light microscopy and the expression of desmin and smooth muscle actin on immunohistochemistry in our study.

Striated muscles were observed in a few specimens of processus vaginalis in all three pathologies and this was statistically significant. The striated muscle believed to develop within the gubernaculum is the cremaster muscle. The striated muscle may have also developed through trans differentiation of the smooth muscle. Innervation appears to play a role in trans differentiation.

The processus vaginalis sacs from four female patients of inguinal hernia did not differ statistically from the processus vaginalis sacs from thirty-three male patients with inguinal hernia with respect to the presence of smooth muscles, distribution of smooth muscle bundles, expression of desmin, smooth muscle actin, presence of striated muscles and presence of myofibroblasts. This contrasts with the results of Tanyel, et al., wherein the authors reported that striated muscles were observed in sacs from girls and not the sacs from boys with inguinal hernia [9]. However, we observed striated muscles in sacs from hydrocele and UDT as well. The study by Tanyel, et al. [9] had not evaluated the other characteristics of the sacs, unlike our study. In both the studies the sample size of girls with inguinal hernia was small. Therefore, larger studies on this aspect may yield a conclusive answer regarding sexual dimorphism of the processus vaginalis sacs.

Processus vaginalis is a special structure with smooth muscle content. If the smooth muscle does not disintegrate, it may hinder the obliteration and give rise to hydrocele and inguinal hernia. The amount of persisting smooth muscle determines the clinical outcome as hernia, hydrocele or UDT. The genitofemoral nerve has been proposed to respond to androgens by release of calcitonin gene-related peptide (CGRP) from its sensory nerve endings to control growth of the gubernaculum to the scrotum. Smooth muscles develop as a result of this signal to help in the descent of the testis. The processus vaginalis obliterates after the descent of testis. The exact mechanism of obliteration of the processus vaginalis is unknown. Failure of the obliteration of the processus vaginalis results in an inguinal hernia, hydrocele or UDT.

Our study highlights the differences in tissue composition in the processus vaginalis sacs in inguinal hernia, hydrocele and UDT, thereby resulting in the varied clinical presentation. Inguinal hernia and hydrocele sacs contain more smooth muscle cells and myofibroblasts and express desmin and SMA in greater magnitude than the sacs from UDT. Newer studies have shown the role of cytokines such as hepatocyte growth factor (HGF) and transforming growth factor beta 1 (TGF-b1) in the closure of processus vaginalis. The presence of HGF receptors in the processus vaginalis may indicate the role of HGF in triggering epithelial-mesenchymal transformation during inguinal hernia closure [14]. TGF-b1 is a potent fibrogenic agent and stimulates fibrosis of processus vaginalis. Mosavi, et al. have observed that the amount of TGF-b1 was higher in communicating hydrocele fluid than in non-communicating hydrocele [15]. Further studies on the role of cytokines may provide insight into the exact mechanism of obliteration of processus vaginalis.

Ours is the first study to analyze the processus vaginalis sacs of inguinal hernia, hydrocele and UDT using histology, immunohistochemistry and electron microscopy simultaneously. We evaluated the presence and maturity of smooth muscle cells using histology and immunohistochemistry and confirmed the presence of myofibroblasts using electron microscopy in all the specimens. We also analyzed the processus vaginalis sacs from female hernia patients; albeit in a smaller number. Due to the implementation of uniform protocol and accurate tests employed to evaluate the stated objectives, our study carried out in a larger sample size adds significantly to the available literature on the characteristics of the processus vaginalis in inguinal hernia, hydrocele and UDT. However, there are a few limitations of our study. The sample size of female inguinal hernia patients was small. Therefore, the validity of our results need to be studied in a larger sample before generalizing the results in this subset. We conducted a qualitative and semi-quantitative analysis on the smooth muscle presence in the processus vaginalis specimens due to technical limitations. In spite of these limitations, our study highlights the differences in the characteristics of the smooth muscle cells in inguinal hernia, hydrocele and UDT which lead to the variation in presentation in these conditions.

## Conclusion

The processus vaginalis sacs in pediatric inguinal hernia, hydrocele and undescended testis differ in the tissue composition. The sacs in inguinal hernia and hydrocele contain smooth muscle cells distributed in bundles which express desmin and SMA. In contrast to this, the sacs from UDT contain sparse smooth muscles, if any. Similarly, the myofibroblasts are present in the sacs of inguinal hernia and hydrocele and rarely found in the sacs of UDT. Between inguinal hernia and hydrocele, smooth muscle bundles and myofibroblasts are present more often in the sacs of inguinal hernia. The clinical presentations in these entities reflect these differences.

## Data Availability

The raw data that support the findings of this study are available from the corresponding author upon reasonable request.

## Abbreviations

UDT: Undescended Testis
GFN: Genitofemoral Nerve
CGRP: Calcitonin Gene–Related Protein
SMA: α-Smooth Muscle Actin
H & E: Haematoxylin and Eosin
ANOVA: One-way analysis of variance
HGF: Hepatocyte growth factor
TGF-b1: Transforming Growth Factor beta 1
